# The Incidence and Risk Factors of Meningitis after Major Craniotomy in China: A Retrospective Cohort Study

**DOI:** 10.1371/journal.pone.0101961

**Published:** 2014-07-08

**Authors:** Chen Chen, Bingyan Zhang, Shenglei Yu, Feng Sun, Qiaoling Ruan, Wenhong Zhang, Lingyun Shao, Shu Chen

**Affiliations:** Department of Infectious Diseases, Huashan Hospital, Fudan University, Shanghai, China; Toronto Western Hospital, Canada

## Abstract

**Background:**

Meningitis after neurosurgery can result in severe morbidity and high mortality. Incidence varies among regions and limited data are focused on meningitis after major craniotomy.

**Aim:**

This retrospective cohort study aimed to determine the incidence, risk factors and microbiological spectrum of postcraniotomy meningitis in a large clinical center of Neurosurgery in China.

**Methods:**

Patients who underwent neurosurgeries at the Department of Neurosurgery in Huashan Hospital, the largest neurosurgery center in Asia and the Pacific, between 1^st^January and 31^st^ December, 2008 were selected. Individuals with only shunts, burr holes, stereotactic surgery, transsphenoidal or spinal surgery were excluded. The complete medical records of each case were reviewed, and data on risk factors were extracted and evaluated for meningitis.

**Results:**

A total of 65 meningitides were identified among 755 cases in the study, with an incidence of 8.60%. The risk of meningitis was increased by the presence of diabetes mellitus (odds ratio [OR], 6.27; *P* = 0.009), the use of external ventricular drainage (OR, 4.30; *P* = 0.003) and the use of lumbar drainage (OR, 17.23; *P*<0.001). The isolated microorganisms included *Acinetobacter baumannii*, *Enterococcus sp*, *Streptococcus intermedius* and *Klebsiella pneumonia*.

**Conclusions:**

Meningitis remains an important source of morbidity and mortality after major craniotomy. Diabetic patients or those with cerebral spinal fluid shunts carry significant high risk of infection. Thus, identification of the risk factors as soon as possible will help physicians to improve patient care.

## Introduction

Meningitis has posed a formidable challenge in neurosurgery, resulting in severe morbidity with a prolonged length of hospitalization, multiple surgeries and higher hospital costs [1]. It is important for surgeons to identify risk factors and provide empirical treatment according to the local surveillance of pathogens once infection is suspected. Thus, the epidemiologic surveillance should be carried out institution by institution.

More than 3000 craniotomies are performed in our clinic annually. Although comprehensive studies have been published [2]–[15], 2 focusing on the evaluation of risk factors related to surgical site infection including meningitis, only few studies considered specific factors for meningitis after major craniotomy [1], [16] 16. The limited data and urgent clinical needs encouraged the conduction of this study. We aimed to investigate the incidence of postcraniotomy meningitis at the department of Neurosurgery in Shanghai Huashan Hospital, to analyze the risk factors involved in the development of meningitis and to identify the etiology agents for empirical treatment.

## Materials and Methods

### Hospital

Huashan Hospital, one of accredited agencies of Joint Commission International (JCI), is a tertiary hospital with 1216 beds and annual admission rate of about 20000 patients. The hospital's neurosurgery department has 67 faculties, 200 inpatient beds and 20 beds dedicated to the neurosurgical intensive care unit, with an average annual admission of 5000 patients.

### Patients

This study retrospectively collected medical records at the Department of Neurosurgery in Huashan Hospital of Fudan University in Shanghai, between 1^st^ January and 31^st^ December, 2008. Patients who discharged during the first 7 days of each month were selected by equidistance cluster sampling. All patients who underwent at least one neurosurgery and survived at least 7 days after surgery were eligible. Patients having only cerebral spinal fluid (CSF) shunt implantations, burr hole trepanations, or stereotactic surgery associated with vascular intervention, transspenoidal or spinal surgery were excluded.

### Ethics statements

All data were anonymously analyzed without individual patient consent due to the retrospective nature of the study. This study protocol was approved by the institutional review boards at Fudan University and Huashan Hospital.

### Data abstraction

Data were abstracted and recorded in a standard form by two investigators and then reviewed in duplicate by another three investigators, all of whom accepted training to familiarize themselves with the performance of the data form at the commencement of the study. We recorded the general information (age, sex, admission number); length of hospitalization; presence of underlying diseases (cardiac failure, chronic obstructive pulmonary disease, diabetes mellitus, chronic renal failure, cirrhosis, connective tissue disease and cancer); Glasgow Coma Scale (GCS); preoperative use of corticoids; preoperative and perioperative use of antibiotics; surgery duration; procedure urgency (elective or emergency); American Society of Anesthesiologists (ASA) score; reasons for surgery (oncological, trauma, vascular, hydrocephalus and others); anesthesia (general, epidural and local); implantation of a foreign body (vascular clips); repeat operations; concurrent infection (lower respiratory tract infection, urinary tract infection, digestive tract infection, blood stream infection and wound infection); invasive operation (tracheal intubation, tracheotomy, mechanical ventilation, external ventricular drainage (EVD), lumbar drainage (LD), urinary catheterization, central venous catheter, topical negative pressure drainage).

### Definition of meningitis

The definition of meningitis must meet at least one of the followings: either 1) patient has organisms cultured from CSF or 2) patient has one of the following signs or symptoms with no other recognized cause: fever (>38°C),headache, meningeal signs and at least one of the followings: a. Increased white cell count, elevated protein, and/or decreased glucose level in CSF. b. Organisms seen on Gram's stain of CSF. c. Positive antigen test of CSF. d. Organisms cultured from blood. e. Diagnostic single antibody titer (IgM) or 4-fold increase in paired sera (IgG) for pathogen [17]. We recorded the date on which diagnostic lumbar puncture was performed or diagnostic antibiotic treatment was used as the date of meningitis.

### Statistical analysis

The data were stored in Epi data, Version 3.1 and were analyzed using SPSS software, Version 20.0. Continuous variables were compared using two-sample *t*-test, while categorical variables were compared using chi-square test. For each of the variables, the odds ratio (OR) was calculated along with a 95% confidence interval (CI) by logistic regression. After the univariate analysis, the independent variables were conducted through multivariate stepwise regression. When the continuous variable, such as the duration of EVD or LD, was shown to be statistically significant associated with postoperative meningitis, we chose suitably coded dummy variables for further analysis.

## Results

### Study population

During the study period, 1162 patients who underwent at least one neurosurgery were sampled. Twenty patients had only CSF shunt implantation; 94 had burr hole trepanation; 75 had vascular interventional surgery; 158 had transspenoidal surgery; 56 had spinal surgery. Two patients died within 7 days after surgery and medical records of 2 patients were incomplete. All of the above 407 were excluded from our study. Seven hundred and fifty-five patients were eligible. The mean hospitalization was 18.65 days (2–69 days) and mean postoperative hospitalization was 6.32 days (1–35 days).

Sixty-five patients (8.60%) were complicated with meningitis. The mean age of patients with meningitis was 40.9±16.4 years; the mean age for those without was 43.8±17.0 years (*P* = 0.179). Forty-one (10.65%) out of 385 men and 24 (6.49%) out of 370 women experienced meningitis (*P* = 0.041). The mean hospitalization for patients with or without meningitis were 28.8±12.1 days and 17.7±8.0 days (*P*<0.001). The mean postoperative hospitalization for patients with and without meningitis were 22.5±10.4 days and 11.8±6.1 days (*P*<0.001). Two patients who developed meningitis died before discharge, resulting in a fatality rate of 0.26%. Neither of them died of meningitis.

### Univariate analysis of risk factors of meningitis after major craniotomy

Variables involved in the univariate analysis are listed in Table 1. Factors with *P* value of less than 0.05 in univariate analysis included male, GCS under 12, EVD, LD, enteral nutrition, surgery duration over 4.5 hours, repeat operations, use of perioperative antibiotics and concurrent infection. Among the cases with concomitant infection, three were surgical site infection, none of which developed meningitis.

**Table 1:** Univariate analysis of risk factors for the development of postcraniotomy meningitis.

Factor	No. at risk	No. of meningitis	Rate of infection (%)	Odds ratio (95% confidence interval)	*P* value
Age (numeric)	755	65	8.60%	0.990 (0.976–1.005)	0.180*
Age greater than 50 years	310	19	6.13%	0.566 (0.325–0.987)	0.045
Male sex	385	41	10.65%	1.718 (1.016–2.906)	0.043
GCS† <12	32	9	28.13%	4.62 (2.033–10.501)	<0.001
Emergency procedure	43	5	11.63%	1.944 (0.785–4.814)	0.151
Diabetes mellitus	27	5	18.52%	2.530 (0.925–6.922)	0.071
Malignancy	10	2	20.00%	2.706 (0.563–13.019)	0.214
Preoperative use of corticoids	40	2	5.00%	0.545 (0.128–2.311)	0.410
Preoperative use of antibiotics	26	2	7.69%	0.416 (0.055–3.118)	0.393
Tracheal intubation	59	9	15.25%	2.057 (0.962–4.401)	0.063
Tracheotomy	18	2	11.11%	1.337 (0.301–5.948)	0.703
Mechanical ventilation	14	3	21.43%	2.987 (0.812–10.990)	0.100
External ventricular drainage	61	16	26.23%	4.680 (2.467–8.878)	<0.001
Lumbar drainage	94	42	44.68%	22.405 (12.535–40.084)	<0.001
Urinary catheterization	725	61	8.41%	0.597 (0.202–1.767)	0.352
Enteral nutrition	71	13	18.31%	2.724 (1.402–5.295)	0.003
Central Venous Catheters	323	25	7.74%	0.822 (0.488–1.388)	0.462
Topical negative pressure	542	48	8.86%	1.120 (0.629–1.996)	0.700
Surgery duration(h)>4.5	286	36	12.59%	2.377 (1.395–4.053)	0.001
Foreign body placement	637	58	9.11%	1.588 (0.707–3.571)	0.263
Repeat operations	13	4	30.77%	4.962 (1.485–16.582)	0.009
Oncological diseases	550	51	9.27%	-	0.895
Trauma	13	1	7.69%	0.815 (0.141–6.399)	0.846
Vascular diseases	111	7	6.31%	0.659 (0.291–1.492)	0.317
Hydrocephalus	5	0	0.00%	-	-
ASA‡ score>2	30	3	10.00%	1.181 (0.348–4.004)	0.789
Use of perioperative antibiotics	523	57	10.90%	3.425 (1.607–3.425)	0.001
Concurrent infection	66	13	19.70%	3.005 (1.539–5.867)	0.001

*For continuous variables, the odds ratio represents that the risk of infection increases n-fold with the change of a unit.

† GCS, Glasgow Coma Scale.

‡ ASA, American Society of Anesthesiologists.

### Multivariate analysis of risk factors of meningitis after major craniotomy

We included all the factors with *P* value of less than 0.2 in the univariate analysis into the logistic multivariate analysis. Table 2 lists all the variables included and remained in the model after multivariate analysis and the *P* value of removed factors. Diabetes mellitus (OR, 6.27; *P* = 0.009), EVD (OR, 4.30; *P* = 0.003) and LD (OR, 17.23; *P*<0.001) were determined as independent risk factors for meningitis after craniotomy. The use of perioperative antibiotics had *P* values between 0.05 and 1. After multivariate analysis, patients using antibiotics had a higher mean age (44.9 years *vs* 40.4 years, *P* = 0.001) and longer surgery duration (4.0 h *vs* 4.4 h, *P* = 0.009).

**Table 2:** Odds ratios for the variables studied by multivariate logistic regression.

Factor	Univariate analysis		Multivariate analysis	
	Odds ratio	*P* value	Odds ratio (95%confidence interval)	*P* value
Age greater than 50 years	0.566	0.045	-	0.523
Male sex	1.718	0.043	-	0.127
Diabetes mellitus	2.53	0.071	6.271 (1.596–24.636)	0.009
GCS*<12	4.62	<0.001	-	0.207
Tracheal intubation	2.057	0.063	-	0.798
External ventricular drainage	4.680	<0.001	4.301 (1.640–11.284)	0.003
Lumbar drainage	22.405	<0.001	17.226 (8.240–36.012)	<0.001
Enteral nutrition	2.724	0.003	-	0.626
Surgery duration(h)>4.5	2.377	0.001	-	0.571
Emergency procedure	1.944	0.151	-	0.140
Repeat operations	4.962	0.009	-	0.257
Concurrent infection	3.005	0.001	-	0.171
Use of antibiotic prophylaxis	3.425	0.001	-	0.098

*GCS, Glasgow Coma Scale.

### Drainage-related infection rate and risk factors of meningitis after major craniotomy

Of the 616 cases without any drain after craniotomy, 16 cases developed meningitis, yielding an incidence of 2.60%. Any CSF drainage led to 20.4-fold (95% CI: 11.1–37.4) increase in the risk of meningitis. For EVD, the increase in risk was 4.7-fold (95% CI, 2.5–8.9); for LD, it was 22.4-fold (95% CI, 12.5–40.1). Compared with catheters without infection, those with infection were more likely to stay longer (EVD, 10.06 days vs. 5.53, *P* = 0.001; LD, 10.21 days vs. 5.73, *P*<0.001). The risk of an EVD- and LD-related infection was significantly increased with longer duration of drainage. Table 3 and table 4 show the effect of drainage duration on the risk of meningitis. After 7 days, the risk of infection for EVD and LD increased by 15.6 (95% CI, 1.8–137.4) and 17.3 (95% CI, 3.4–88.4), respectively.

**Table 3:** Logistic regression model with variables influencing the risk on EVD-related infection.

	No. at risk	No. of infection	Rate of infection	Odds Ratio	95% Confidence interval	*P* value
EVD* duration ≤3days	18	1	5.56%	-	-	0.019†
3 days < EVD duration ≤7 days	20	4	20.00%	4.250	0.428–42.187	0.217‡
EVD duration >7 days	23	11	47.83%	15.583	1.768–137.361	0.013‡

*EVD, External ventricular drainage.

† *P* value represents odds ratios are not consistent among three groups.

‡ The odds ratios are the results of comparison with EVD duration≤3days.

**Table 4:** Logistic regression model with variables influencing the risk on an LD-related infection.

	No. at risk	No. of infection	Rate of infection	Odds Ratio	95% Confidence interval	*P* value
LD* duration≤3days	22	2	9.09%	-	-	0.001†
3days< LD duration≤7days	22	7	31.82%	4.667	0.846–25.753	0.077‡
7days< LD duration≤14days	30	19	63.33%	17.273	3.377–88.356	0.001‡
LD duration >14days	20	14	70.00%	22.333	4.096–132.932	<0.001‡

*LD, Lumbar drainage.

† *P* value represents odds ratios are not consistent among three groups.

‡ The odds ratios are the results of comparison with EVD duration ≤3days.

**Figure 1:**
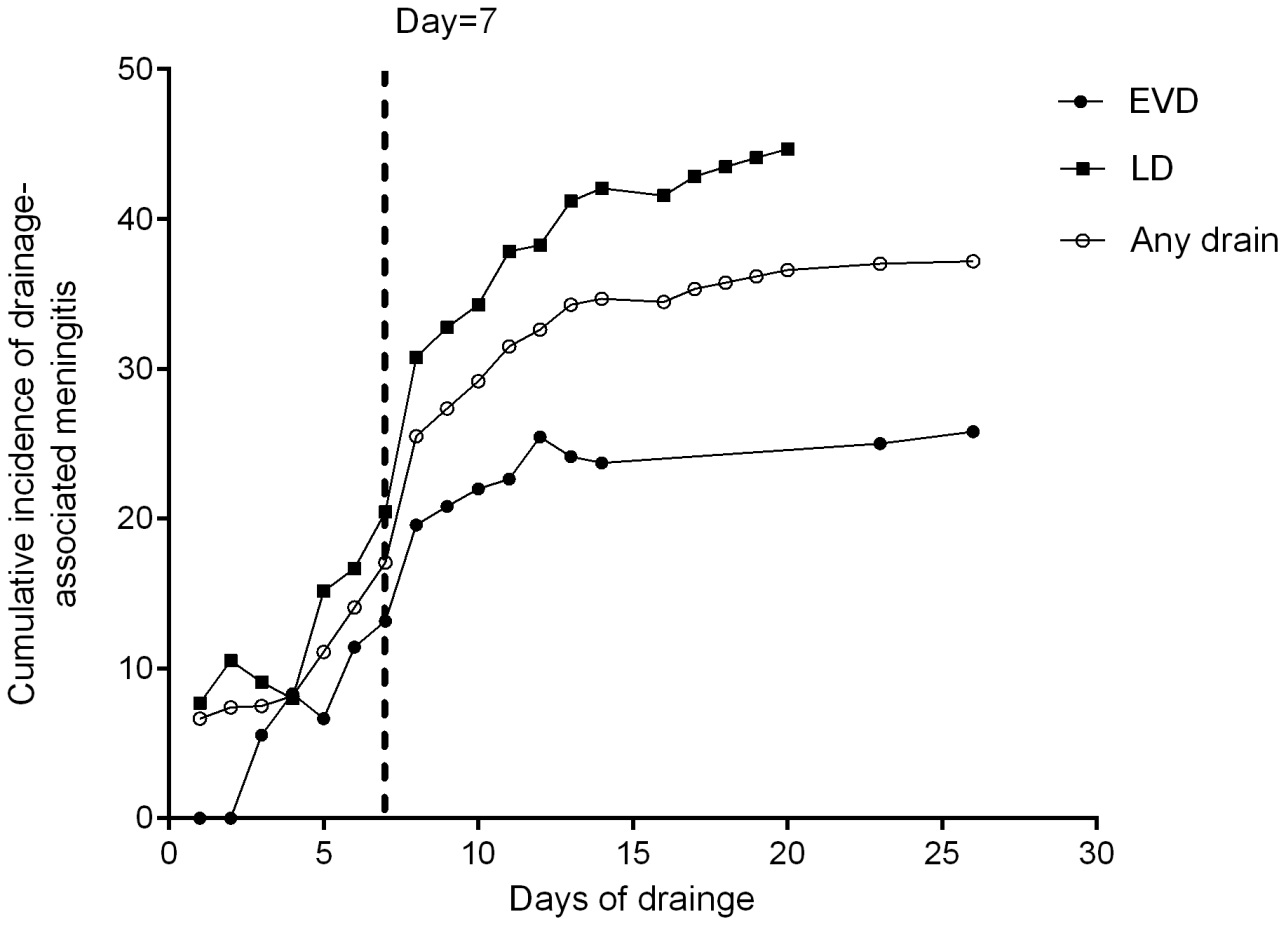
Cumulative risk of drainage-associated meningitis. EVD, external ventricular drainage; LD, lumbar drainage; Any drain, data included patients either with EVD or with LD.

The cumulative infection rate of meningitis related to EVD and LD is depicted in Figure 1. The risk increased during the first 12 days in patients with EVD and the infection rate reached plateau after then. In LD, cumulative rate of infection increased in the first 13 days. The risk of meningitis in LD was much higher than the one in EVD.

### Microbiology

Among the 65 cases with infection, 48 CSF samples from 44 patients (67.7%) were sent for detection of pathogens. Positive cultures were only encountered in five of them, yielding a positive rate of 10.4%. The offending organisms were two *Acinetobacter baumannii*, one *Enterococcus sp*, *Streptococcus intermedius* and *Klebsiella pneumonia*.

## Discussion

The incidence of postneurosurgical meningitis was about 0.3%–8.9% in previous reports [1], [5], [7], [8], [10], [12], [14]–[16], [18], [19], only two of which are comparable to ours because of the similar inclusion criteria for the surgery type [1], [16] 21. In this study, the incidence of meningitis was 8.6%, a little higher than those reports that were focused on craniotomy (0.8%–1.5%) [20]. This higher incidence may be due to the different patient population and constitution of the diseases. Given that the department of neurosurgery at Huashan Hospital is one of the largest volume centers in Asia and the Pacific and severe patients from across China are referred for medical treatment [21], [22], surgeries are usually challenging, demanding and time-consuming, resulting in a higher rate of infection.

Based on review of the literatures, the positive rate of CSF culture varied among 32%–94% [1], [12], [16], [19], [23]. Compared with these studies, positive microbiology in our series was lower. We consider the situation could be closely related to the use of antibiotics. Prophylactic antibiotic is widely used in our country. Among the patients with meningitis, 57 were administered perioperative antibiotics, which was 87.7% of the infected population. Besides, a total of 48 CSF samples from 44 patients were sent for culture which accounted for only 67.7% of the cases. This also indicated that only four patients had multiple cultures. These two reasons mainly resulted in this low microbiology. We strongly suggest that patients with high suspicion of meningitis should conduct CSF culture more frequently, considering positive result is of great clinical essence.

Gram-negative organisms predominated as meningitis pathogens in our cohort. Although *A. baumannii* is less common compared to Staphyloccoci, it has emerged as one of the most troublesome pathogens for healthcare institutions worldwide over the last 15 years [24]. Furthermore, in several studies, especially in those published after 1992, gram-negative rods and Enterobacteriaceae have played an important role [1], [7], [8], [14]–[16], [18], [25]–[28]. Thus, our finding can provide some information for empirical treatment in our institute.

The independent risk factors that were identified in our study by multivariate logistic analysis include diabetes mellitus, EVD and LD, of which LD had the highest OR. The longer duration of LD represents increased risk of meningitis. Especially, surgeons should pay special attention to patients with shunts indwelled between day 4 and day 13. This finding is similar to Scheithauer's research, in which they found that LD-associated infection rate is highest between days 4 and days 9 of LD [29]. Due to the limited number of cases with LD in both studies, our study just reconfirmed their findings.

Craniotomy with external drainage has a higher risk of infection [2], [7], [9], [10], [14], [16], [27]. Our study also confirmed a strong relation between the duration of EVD and meningitis. Patients with infection experienced significantly longer catheterizations than did their uninfected counterparts. The data also showed an increasing risk of infections during the first 10 days of catheterization, after which the infection rate became steady.

Generally, LD has been considered to be safer than EVD [30]. However, our study suggests otherwise. The cause is currently not clear to us. Scheithauer *et al* provided a plausible explanation. It may be due to the higher risk of contamination associated with the internal position of the LD compared to the external position of the EVD [29].

Patients with diabetes in our cohort have a significant higher potential to develop meningitis than those without. Generally, patients with diabetes who underwent elective surgeries had to maintain blood glucose under 11.0 mmol/L in our cohort. It is believed that diabetic patients are prone to infection due to impaired immunity. The presence of diabetes itself would predispose to infection. So whether a stricter blood control would bring benefits to these patients is uncertain, which is awaiting further study. In all, our result indicates that surgeons and nurses should pay close attention to the patients with this underlying disease before and after surgery.

We did not find that the use of perioperative antibiotics had any protection against meningitis. On the contrary, in univariate analysis, antibiotic prophylaxis played a role as a risk factor. This may be attributed to the advanced age and longer duration of surgery in patients with antibiotics, indicating that the diseases are more serious and surgery more complicated. Whether antibiotic prophylaxis is effective is in enduring dispute. Barker *et al* conducted a meta-analysis involving six prospective randomized trials or trial subgroups [31] 37. The pooled odds ratio was 0.43 with a *P* value less than 0.5. Accordingly, this research suggested modest benefit of antibiotic in preventing meningitis after craniotomy.

The limitation of our study is the fact that it is retrospective and depends on the accuracy of the data in clinical charts, resulting in selection bias. In addition, we did not include some studied factors, such as CSF leakage, multiple catheter insertion of CSF shunts. Their contributions to meningitis are impossible to evaluate.

In conclusion, meningitis remains an important source of morbidity and mortality after craniotomy. Here, we determined the incidence of meningitis, risk factors and microbiology that can cause infection with a high frequency in our hospital. Thus, identification of the risk factors for meningitis and use of empirical treatment as soon as possible may improve patient care.
